# Supplementary material for: Best Practices for the Management of Patients with Non-Tuberculous Mycobacterial Pulmonary Disease According to a German Nationwide Analysis of Expert Centers
Source: Healthcare (Basel). 2023 Sep 22;11(19):2610. doi: 10.3390/healthcare11192610 (PMC10572995; doi:10.3390/healthcare11192610)
Supplement: Supplementary file 1 [file healthcare-11-02610-s001.zip › healthcare-2380242-supplementary.pdf]

Best practices for the management of patients with non-tuberculous mycobacterial pulmonary disease according to a German nationwide analysis of expert centers

Supplementary tables

Table S1: Best practices in the NTM-PD treatment process presented by management phase

**Management phase**	**Element**	**Description**
Referral	Direct patient transfer to specialized centers	• Due to the flexible structures of a specialized outpatient care [1], patients can present without a referral from a pulmonary specialist.<br>This facilitates primary physician contact for patients as resident pulmonologists usually have long waiting lists. In the clinic, patients have to wait about 2 weeks for an appointment.
Diagnosis	Performance of regular sputum test	• In cystic fibrosis and bronchiectasis patients, a sputum sample is tested for NTM at least once a year as standard (i.e., once per year in the high risk population or in case of typical NTM-PD symptoms, such as increase in coughing and amount of sputum, weight loss and fever/night sweats; bronchoalveolar lavage (BAL) should be tested for acid fast bacilli (AFB) in suspected cases) [2,3].
	Use of HRCT images for higher precision in the diagnosis	• HRCT images can be obtained in any radiological department with multidetector CT facilities. Standard slice thickness required for HRCT is 1 mm with the possibility for multiplanar reconstruction [4].
	Use of established communication channels for queries (by phone call or email) during the diagnostic process to enable correct and guideline-based diagnosis of NTM-PD	• Using emails in the diagnostic process either internally between departments or even externally with other centers can result in a quicker and more precise diagnosis.<br>• Telephone calls with external specialist departments outside of the clinic can enable rapid therapy decisions and can as well have an effect on the outcomes of the therapy.
Therapy	Using medical reports (*Arztbriefe* in German) to share recommendations of action to the referrer	• Physician's letters are written to determine the next steps for the patients including next steps of action, such as recommendations for referral, diagnosis and therapy and recommendations after diagnosis/start of therapy.
	Individual hotline for better support of patients. Patients will be able to make telephone calls at any point in time with their questions concerning the therapy	• Individual support and answering telephone inquiries from patients at any time.
Follow-up	Follow-up of patients for regular check-ups every 3 to 6 months	• After initiation of the therapy, the patient is revisited every 3 to 6 months, depending on their condition, comorbidities, etc., to monitor the course and manage the underlying disease.<br>• Patients who are not on acute treatment are seen every 6 months for follow-up.

AFB, acid fast bacilli; BAL, bronchoalveolar lavage; CT; computer tomography; HRCT, high-resolution computed tomography; NTM-PD, non-tuberculous mycobacteria pulmonary disease.

Table S2: Possible best practices in the NTM-PD treatment process presented by management phase

Management phase	Element	Description
Referral	An information flyer for the referring and/or treating physicians to gain more knowledge on NTM-PD	• An information flyer dealing with NTM-PD diagnosis and therapy is potentially helpful in raising awareness among both referring physicians as well as treating physicians.<br>The distribution of these flyers could, for example, be combined with the forwarding of laboratory findings to the treating physicians.
	NTM-PD awareness activities combined with information activities for tuberculosis	• One opportunity might be to link NTM-PD awareness campaigns to current promotional activities on the subject of tuberculosis. In this way, a larger audience could be reached, and the awareness of NTM-PD could be broadened.
Diagnosis*	Establishment of protocols (SOPs etc.) to support the diagnostic process	• Establishing protocols (SOPs, etc.) for NTM-PD management, e.g., using a checklist for the main physician can easily be implemented and help speeding up the differential diagnosis. This can especially be helpful when key positions are reassigned, and experienced colleagues leave the department.
Therapy	Focus on patient empowerment, either through homework, training, or knowledge sharing (such as publications) to raise treatment engagement	• Offering educational training to the patients to raise awareness and offer better support. Patients may be able to ask more meaningful questions and receive more useful answers.<br>• The patient might be given a diary to obtain all existing clinically relevant findings and in order to keep track of the result reports.<br>• Alternatively, publications in lay language could be an efficient and easy way to educate the patients.
Follow-up	A cross-center bronchiectasis and/or NTM-PD registry for monitoring of the patient population	• The establishment of a registry for patients with NTM-PD in the region would make it possible to collect and evaluate valuable patient data (data analysis might be performed in the context of a doctoral thesis to improve patient management and disease control). Data from other locations and across laboratories might also be included which would offer the possibility to share knowledge and learnings across centers.

**All centers discussed SOPs and only 1 center expressed a wish for an establishment of such protocols.*

NTM-PD, non-tuberculous mycobacteria pulmonary disease; SOP, standard operating procedure.

Table S3: Challenges/barriers in the NTM-PD treatment process presented by management phase

Management phase	Element	Description
Referral	Limited experience from the referring physician (including general practitioners and pulmonary specialists) with the diagnosis and treatment of NTM-PD	• Referring physicians often have little experience with the diagnosis and treatment of NTM-PD, which can lead to delays in initiating therapy and underestimation of the patient's risk.
	No specific request for investigation of NTM-PD infection during transferal leading to most patients being incidental findings	• Patients very rarely come with a specific request for 'investigation of NTM-PD infection', as this would complicate the referral process.
	Incomplete documentation of findings and result reports leading to increased effort and complications	• The patient's findings are often incomplete, which can prolong the diagnosis time, since telephone consultation for clarification with the referring physicians is very time-consuming.<br>• The main problem for the clinic in connection with patient findings is the lack of HRCTs, which are needed for a correct diagnosis.
	Referral is only possible through a respiratory specialist, which can lead to extended waiting times	• Patients usually present at the clinic on referral from established pulmonary specialists and doctors from other hospitals. Due to existing health insurance structures, a referral is only possible through a respiratory specialist, leading to long waiting times of up to 9 months.
Diagnosis	Lack of time and staff to make diagnostic and treatment decisions, as the disease is individual and complex	• Since the diagnosis and treatment decision is individual, complex, and multifactorial, it can be very lengthy.<br>• There is a lack of time and staff, e.g., to discuss cases in detail and to brief new colleagues.
Follow-up	Regular feedback with the referring physician	• Since the complete microbiological analysis (sub-species; sensitivity testing) requires about 6 weeks, a change in therapy may be necessary at some point.<br>• Regular feedback with the referring physician would be very important, but at the same time highly time-consuming.
	Lack of adherence to guideline-based therapies and potentially specified therapy plans.	• Due to the frequent lack of NTM-PD expertise, adherence to guideline-recommended therapies is low. Some patients might be treated incorrectly, and some therapies might be discontinued.

HRCT, high resolution computed tomography; NTM-PD, non-tuberculous mycobacteria pulmonary disease.

Table S4: Best practices in center infrastructure

Section/aspect	Element	Description
Organizational structure	Internal lung boards (specialist conferences) for better communication and training, e.g., in the diagnostic process to discuss unclear cases	• When findings are analyzed during NTM-PD diagnosis, unclear/special NTM-PD cases can be discussed in daily specialist conference called a lung board.<br>This is desirable to get an overview of the patient's individual situation and to enable guideline-compliant therapy.
	An affiliation of a specialized outpatient care	• All centers that were analyzed offer outpatient and inpatient care for patients with NTM-PD for the clarification of a possible initial NTM-PD diagnosis.<br>• A specialized outpatient care is crucial for NTM-PD patient management and can lead to fast and efficient NTM-PD diagnosis, therapy and follow-up.
	Internal electronic recording of patient history (*Electronic Recording System*) to go directly into the hospital information system	• Electronic recording of the patient history (if not already known by the hospital) with all available findings. The current medication and findings brought along by the referring physician will be entered directly into the hospital systems. The patients usually bring findings (e.g., CTs, medical reports, etc.) to the outpatient clinic. Cystic fibrosis patients often bring their own sputum sample if they can produce sputum.
	Merging of tuberculosis and NTM-PD specialized outpatient care	• The establishment of a new tuberculosis/NTM-PD specialized outpatient care enables better medical care for patients and facilitates the billing of NTM-PD-related services.<br>• By merging tuberculosis and NTM-PD specialized outpatient care centers, case numbers per quarter can be easily assessed.
Communication	Tele-radiology as part of regular NTM-PD boards	• Tele-radiology as part of regular, e.g., monthly NTM-PD boards for excellent communication across different disciplines, such as respiratory specialists, infectious disease specialists, microbiologists, radiologists, and if needed pharmacists and thoracic surgeons for complex cases.
	Radiologist to exchange images directly via an online interface to streamline processes	• Radiologists have the opportunity to interface directly with the clinic to share images online which streamlines the process.
Finance	Documentation of all services rendered and forwarding of these to the medical administration department	• Unlike in the DRG (Diagnosis Related Groups) system, services can be listed and billed individually.<br>• The doctor documents all services rendered and forwards them to the medical administration department.

CT, computer tomography; DRG, Diagnosis Related Groups; NTM-PD, non-tuberculous mycobacteria pulmonary disease.

Table S5: Possible best practices in center infrastructure

Section/aspect	Element	Description
Organizational structure	Implementation of nationwide training events to share awareness and engage referrers and especially GPs as they have a key role as 'entry gatekeeper'	• A national/cross-regional event to share insights, generate awareness for NTM-PD or generally rare lung diseases and to engage referrers, in particular, GPs. It could be a requirement for specialized outpatient centers to host such events, including expert rounds (expert lectures of the participating institutions) to provide education on a regular basis in a regional setting.<br>• The 'family doctor' takes on a more significant role in NTM-PD patient management due to the doctor being an 'entry point' and first point of contact for patients prior to initial diagnosis which makes good integration and communication essential. The goal would be that patients are earlier referred to the pulmonary specialist clinic for clarification of NTM-PD.<br>• The information and learnings could also be shared through publications targeting GPs.
Communication	A virtually-shared network for referring physicians and clinicians to exchange patient information would save efforts and time also in cases where second opinions are required	• A shared network for patient information would save a lot of time and organizational effort.<br>• The establishment of a digital platform (e.g., dedicated line for data transfer) between practices, hospitals, and clinics for the digital sharing of findings could save efforts and time and could as well improve the process of sharing patient data when needed for second opinions.
	A specialized team consisting of few dedicated physicians and caregivers with clear responsibilities for patient care to avoid confusion of external communications around the patient history, ease the effort and time spent on internal alignment, and offer better patient support	• The communication between the involved parties is called 'whisper mail' due to several doctors not aligning with each other on the findings. It is therefore difficult to know, e.g., which details have already been discussed or which findings have already been made by other colleagues.<br>• With fewer people involved it is possible to facilitate the transfer of information. If most patients refer only to one of two physicians for the management side effects; regular follow-ups, etc., the primary care physician would have to align with fewer colleagues.<br>• Fewer involved parties would also optimize and simplify the patient support. A possible solution could also be to implement a patient support program through an external nurse serving as main point of contact for the patient. The nurse could help the patients during the therapy, e.g., offer side effect management or help with device handling.
	Use of an app as a communication tool among healthcare professionals	• For example, the Siilo App is a free secure messaging app for healthcare professionals to discuss patients and protocols easily and securely with colleagues through messages, voice / video calls, photos, and videos without interrupting the daily work. It can be used as a tool for communication with colleagues, but also, as a networking tool.
Finance	Better explanation and filing of previous findings to possibly save some costly investigations	• The NTM-PD-specific services are billed using a mixed calculation.<br>• In the inpatient setting, the DRG code for tuberculosis (E76C) is used for patients with NTM-PD for the first 14 days, irrespective of the X-ray, CT or bronchoscopy performed.

		• Based on better information and the provision of preliminary findings, some examinations could possibly be avoided.

CT, computer tomography; DRG, Diagnosis Related Groups; GP, general practitioner; NTM-PD, non-tuberculous mycobacteria pulmonary disease.

Table S6: Challenges/barriers in center infrastructure

Section/aspect	Element	Description
Communication	Lack of a suitable external communication channel for the exchange of patient data, findings, and diagnoses	• There is no suitable channel for the exchange of patient data and findings outside of lung boards.<br>• Communication with resident colleagues currently occurs via patients (and medical reports).<br>• For questions, the doctors in the outpatient clinic have to call the clinic hotlines, with long waiting times or, no response.<br>• An interdisciplinary digital portal should be implemented to discuss and share patients' data.
Finance	Complex and time-consuming processes for services billed both in the inpatient and outpatient setting which can lead to mistakes	• Not all services billed in the inpatient setting are reimbursed to cover costs and must therefore be billed with the help of a mixed calculation/cross-financing, which is complex and time consuming.<br>• Difficulty of billing all NTM-PD-relevant services (e.g., bronchoscopy) in the outpatient setting via the uniform evaluation standard ((*Einheitlicher Bewertungsmaßstab* in German) – it is time consuming and can lead to mistakes.
	Almost no in-house expertise available for billing via the uniform evaluation standard (*Einheitlicher Bewertungsmaßstab* in German) despite a very complicated system	• At present, there is almost no in-house expertise available for billing via the uniform evaluation standard (*Einheitlicher Bewertungsmaßstab* in German) and it is unclear who is responsible (general administration/medical administration).<br>• In addition, the uniform evaluation standard (*Einheitlicher Bewertungsmaßstab* in German) system is 'highly complicated'.

NTM-PD, non-tuberculous mycobacteria pulmonary disease.

Table S7: Best practices (BP) and possible best practices (PBB) that were selected or rejected after the transversal analysis

Best practice and possible best practice elements selected or rejected after the transversal analysis (N=24)				
a. Clinical phases in the management of NTM-PD				
Element no.	Referral			
1	I	Direct patient transfer to specialized centers after patient referral	BP	Selected
2	II	An information flyer for the referring and/or treating physicians to gain more knowledge on NTM-PD	PBP	Selected
3	III	NTM-PD awareness activities could be combined with information activities for tuberculosis	PBP	Selected
	Diagnosis			
4	I	Performance of regular sputum test (i.e., once per year in high risk populations or in case of typical NTM-PD symptoms, such as increase in coughing and amount of sputum, weight loss and fever/night sweats; bronchoalveolar lavage (BAL) should be tested for acid fast bacilli (AFB) in suspected cases) [2,3]	BP	Selected
5	II	Use of high-resolution computed tomography (HRCT) imaging for higher preciseness in the diagnosis	BP	Selected
6	III	Use of established communication channels for queries (by phone call or email) during the diagnostic process to enable correct and guideline-based diagnosis of NTM-PD	BP	Selected
7	IV	Establishment of protocols to support the diagnostic process	PBP	Selected
	Therapy			
8	I	Use of medical reports (*Arztbriefe* in German) to share recommendations of action to the referrer	BP	Selected
9	II	Focus on patient empowerment, either through homework, training, or knowledge sharing (such as publications) to raise treatment engagement	PBP	Selected
10	III	Individual hotline for better support of patients. Patients will be able to make telephone calls at any point in time with their questions concerning the therapy	BP	Rejected
	Follow-up			
11	I	Follow-up of patients for check-ups every 3 to 6 months	BP	Selected
12	II	A cross-center bronchiectasis and/or NTM-PD registry for monitoring of the patient population	PBP	Selected
B. Infrastructure in the NTM-PD management				
	Organizational structures			
13	I	Internal lung boards (specialist conferences) for better communication and training, e.g., in the diagnostic process to discuss unclear cases	BP	Selected
14	II	An affiliation of a specialized outpatient care	BP	Selected

15	III	Implementation of nationwide lung boards (training events) to share awareness and engage referrers and especially GPs due to their key role as ‘entry gatekeeper’	PBP	Selected
16	IV	Merging of tuberculosis and NTM-PD specialized outpatient care	BP	Rejected
17	V	Internal electronic recording of patient history (*Electronic Recording System*) to go directly into the hospital information system	BP	Rejected
	Communication			
18	I	Tele-radiology as part of regular NTM-PD boards (composed of experts from different disciplines, such as respiratory specialists, infectious disease specialists, microbiologists, radiologists, and if needed pharmacists and thoracic surgeons for complex cases)	BP	Selected
19	II	Exchange of images by radiologists directly via an online interface for effort and time savings	BP	Selected
20	III	A virtually-shared network for referring physicians and clinicians to exchange patient information would save efforts and time also in cases where second opinions are required	PBP	Selected
21	IV	A specialized team with clear responsibilities for patient care to avoid confusion of external communications around the patient history, ease the effort and time spent on internal alignment, and offer better support to the patient	PBP	Selected
22	V	Use of an app as a means of communication between medical specialists	PBP	Rejected
	Finance			
23	I	Better explanation and submission of previous findings to possibly save some costly investigations	PBP	Selected
24	II	Documentation of all services rendered and forwarding of these to the medical administration department	BP	Rejected

AFB, acid fast bacilli; BAL, bronchoalveolar lavage; BP, best practice; HRCT, high-resolution computed tomography; GP, general practitioner; NTM-PD, non-tuberculous mycobacteria pulmonary disease; PBP, possible best practice.

References

1. G-BA. Richtlinie des Gemeinsamen Bundesausschusses über die Ambulante Spezialfachärztliche Versorgung nach § 116b SGB V. 2022. Available online: https://www.g-ba.de/richtlinien/80/ (accessed on 25 November 2022).
2. Polverino, E.; Goeminne, P.C.; McDonnell, M.J.; Aliberti, S.; Marshall, S.E.; Loebinger, M.R.; Murris, M.; Canton, R.; Torres, A.; Dimakou, K.; et al. European Respiratory Society guidelines for the management of adult bronchiectasis. *Eur Respir J* **2017**, *50*, doi:10.1183/13993003.00629-2017.
3. National Guideline, A. National Institute for Health and Care Excellence: Guidelines. In *Cystic Fibrosis: Diagnosis and management*; National Institute for Health and Care Excellence (NICE) Copyright © NICE 2017.: London, 2017.
4. Biederer, J.W., J. E.; Bolte, H.; FInk, C.; Tuengerthal, S.; Rehbock, B.; Hieckel, H. G.; Diederich, S.; Hofmann-Preiss, K.; Lörcher, U.; Heussel, C. P. Protocol Recommendations for Computed Tomography of the Lung: Consensus of the Chest Imaging Workshop of the German Radiologic Society. **2008**.

Interview guides (German), used by data collection agency

Interview guides, specialised by hospital department.

INTERVIEW GUIDE ZUR BEGLEITUNG DER OBSERVATIONEN VON NTM ZENTREN IN DEUTSCHLAND
- FACHBEREICH: CONTROLLING/ ADMINISTRATIVE ABTEILUNG -

Vorstellung und Eröffnung des Interviews

- Vorstellung von ALCIMED; kurze Beschreibung des Projektes
- Können Sie bitte Ihre derzeitige Position kurz erläutern?
  - Wofür sind Sie genau zuständig?
- Ist Ihnen NTM, die nicht-tuberkulösen Mykobakterien, ein Begriff?
  - Wenn nicht, Alternativen anbieten:
    - CF mit atypischer Infektion
    - BE mit zusätzlicher Infektion mit NTM
    - Atypische Lungeninfektionen (MAC etc.)
- Haben Sie Erfahrung mit der Abrechnung bzw. dem Management von NTM-spezifischen Unterlagen?
  - Wie sieht diese genau aus?

Patientenzahlen

Für die bestmögliche Beantwortung einiger der folgenden Fragen, werden den Kollegen vom Controlling einige Fragen zuvor per Email zugeschickt.

- Gibt es bei Ihnen eine Ambulanz, wo NTM Patienten behandelt werden?
- Gibt es auch eine für NTM Patienten spezifische Ambulanz (NTM Ambulanz)?
  - Wie sieht deren Organisationsstruktur aus? (ASV, etc.)
- Wie viele NTM Patienten behandelt dieses KH pro Monat bzw. pro Jahr?
  - Unter welcher Ziffer werden diese Patienten abgerechnet? (A31.0?)
- Im Vergleich dazu: Wie viele Tuberkulose Patienten behandelt dieses KH pro Jahr?
- Wie viele NTM Patienten werden rein ambulant behandelt und welcher Anteil wird stationär aufgenommen?
- Wie viele NTM Hauptdiagnosen (A31.0) werden gestellt, bzw. wie viele Nebendiagnosen?
  - Hat sich dies in den letzten Jahren geändert? Gibt es in letzter Zeit mehr Patienten mit einer NTM Diagnose?
  - Was könnte der Grund dafür sein?
    - Stärkung des Bewusstseins; mehr Aufklärung über NTM?

Offene Frage:

- Gibt es derzeit Probleme im Abrechnungsprozess von NTM-spezifischen Leistungen?
  - Wenn ja: Welche wären dies konkret?

Management & Abrechnung von NTM Leistungen

- Wie ist der administrative Ablauf NTM-spezifischer Abrechnungen bei Ihnen organisiert?

    - Gibt es bei Ihnen Experten, welche sich auf ein bestimmtes Themengebiet zur Leistungsabrechnung spezialisiert haben?
    - Benutzen Sie für die Abrechnung besondere Hilfsmittel? (Software etc.)
- Sind NTM-spezifische Abrechnungen besonders anspruchsvoll? Warum?
- Unterscheidet sich die Abrechnung von NTM Patienten im Vergleich zu anderen Lungen-spezifischen Infektionskrankheiten, wie z.B. Tuberkulose oder andere?
- Woher beziehen Sie Ihre Informationen zu den verschiedenen Prozeduren und Tests bzgl. der Diagnose und Behandlung von NTM Patienten in der Klinik?
    - Wie validieren Sie diese Informationen?

Offene Diskussion:

- Wo sehen Sie derzeit Probleme hinsichtlich der Abrechnung und der Organisation von NTM Patienten? Wie sähen konkrete Ideen für eine Verbesserung aus? (z.B. Schulung?)

Organisationsstruktur

- Hat ihre Abteilung administrative Werkzeuge entwickelt oder nutzt eine bestimmte Software, welche bei der Abrechnung hilft?
    - Gibt es z.B. eine Datenbank? Für Tb- oder NTM Patienten?
    - Gibt es konkrete Ideen oder Bedürfnisse hinsichtlich einer Unterstützung?
- Haben Sie oder Ihre Kollegen ein spezielles Training oder Unterstützung erhalten für die Abrechnung von NTM-spezifischen Leistungen, wie Labor-, radiologische und pulmologische Untersuchungen?
    - Bestünde daran Interesse? Welche Themengebiete sollten besonders geschult werden?
        - Training im kodieren der Untersuchungen zu verschiedenen Diagnosen
        - Für die Qualitätssicherung von abzurechnenden Leistungen
        - Optimierung der Abrechnung für eine bessere Vergütung für NTM Leistungen
- Ist das KH Personal geschult oder angewiesen, NTM Patienten immer unter der Diagnose NTM zu kodieren (ICD10: A31.0) für die Vergütung?
    - Wenn nicht, worunter dann?
    - Was wäre der Grund für die Abrechnung unter einer anderen ICD Kodierung?
        - Eine bessere Vergütung? Welche Codierung wird dann angegeben?
- Wie häufig wird die Diagnose A31.0 vergeben als
    - Erstdiagnose?
    - Zweitdiagnose?
    - Welche Konsequenz hat die Codierung A31.0 als Erst- bzw. Zweitdiagnose?
    - Hat das Controlling einen Einfluss auf die Codierung von NTM Untersuchungen, welche von den klinischen Kollegen angegeben werden?

Kommunikation mit anderen Fachabteilungen

- Wie ist die momentane Kommunikation mit den klinischen Kollegen?
    - Wie häufig und mit wem wird kommuniziert?
        - Chefarzt, Bereichsleiter; Andere?

        - Kommentar Insmed/TL: Mit dem medizinischen Kodierungsexperten oder DRG Beauftragten
    - Wie findet dieser Austausch von Informationen statt?
        - Email, Telefon; persönlicher Kontakt?
- Gibt es einen regelmäßigen Austausch von Informationen?
    - Von welcher Seite geht dieser Kontakt meist aus?
    - Welche Informationen werden regelmäßig angefragt? Oder wird nur kommuniziert, wenn offene Fragen bestehen?
    - Besteht Verbesserungsbedarf? Wie sähe dieser konkret aus?

Vergütung von NTM-spezifischen Untersuchungen

- Nach welchem Versorgungskonzept rechnet dieses KH NTM-spezifische ambulante Leistungen ab?
    - ASV (Ambulante spezialfachärztliche Versorgung; in Bayern gibt es nur 1 ASV)
        - Wenn keine ASV vorhanden ist:
            - Wieso wurde bisher keine ASV im KH beantragt?
            - Gibt es Pläne, dies zukünftig zu beantragen?
        - Gab es schon Probleme bei personengebundener ASV, wenn Mitarbeiter die Abteilung verlassen haben?
        - Wie wurden bzw. würden sie in Zukunft solche Probleme behoben bzw. beheben?
    - Persönliche Ermächtigung (PE)
        - Für welche Art von Patienten ist eine PE vorhanden?
        - Ist die Anzahl von Patienten in der PE eingeschränkt?
        - Was wären die Auswirkungen, wenn sich die Patientenzahlen (z.B. für NTM) stark erhöhen würden?
    - MVZ
    - Andere ? (Hochschulambulanz; Spezialambulanz?)
- Wie unterscheiden sich die verschiedenen Abrechnungsmethoden voneinander?
    - Was sind die bestehenden Stärken und Schwächen?
- Seit wann rechnen Sie nach diesem Vergütungssystem ab?
    - Wird dieses System als positiv empfunden bzgl. Zeit und Aufwand der Abrechnungen?
    - Auch spezifisch für die Abrechnung von NTM Leistungen?
- Wie viel Vergütung erzielt eine “ICD-10:A31.0“ Diagnose?
    - Wenn nicht zuvor beantwortet: Wie sehen die Unterschiede aus, wenn NTM als Erst- bzw. als Zweit-Diagnose gestellt wird?
- Wer kodiert die NTM-spezifischen Leistungen?
    - Der Arzt bzw. der Pulmologe: Hat dieser eine DRG Schulung bekommen?
    - Oder Kollegen vom Controlling?
    - Ist die Kodierung von NTM Leistungen zu Ihrer Zufriedenheit oder ist es sehr zeit-bzw. arbeits-intensiv, alle benötigten Informationen für die Abrechnung zu bekommen?
- Nach welchen Kriterien wird kodiert?
    - Finden alle Diagnosen eine Berücksichtigung in der Kodierung?

- Wenn ja: Nach welcher Abstufung bezgl. der Haupt- und Nebendiagnosen?
- Wenn nein: Warum wird nicht alles berücksichtigt?
- Gibt es Fälle, bei der die Diagnose für NTM (A31.0) nicht angegeben bzw. kodiert wurde, obwohl NTM diagnostiziert wurde? (NTM Patient)
  - Worin liegt da der Grund?

- Inwieweit können mit der NTM Kodierung die Kosten der NTM Leistungen gedeckt werden?
  - Wie sehen die Unterschiede in der Vergütung bei der Abrechnung ambulanter Patienten (EBM Ziffer; ASV etc.) und stationärer Patienten (DRG Satz) aus?
  - Wie sehen diesbezüglich Unterschiede bei GKV bzw. PKV Patienten aus?
- Haben Sie Einsicht, welche NTM Leistungen kostendeckend oder sogar gewinnbringend sind, bzw. welche nur unzureichend abgerechnet werden können, so dass Verluste verbucht werden?
  - Wenn ja: Welche sind dies konkret bzw. in welchen Prozessen? (Diagnose, Behandlung, Überwachung)
    - Sputum, Biopsien, Tracheobronchialsekret, Abstriche, Blut, Liquor, etc.
    - Resistenztestungen, Bestimmung der Subspezies etc.
    - Ultraschall-, CT-, Röntgen-Untersuchungen; Lungenfunktionstests; Bronchoskopie etc.
    - Andere?
  - Ist es möglich, bei unzureichend abrechenbaren Leistungen, den Patienten sektorübergreifend abzurechnen? Wie genau sähe dies aus?
- Wie hoch ist die Vergütung der KK und welche Kosten entstehen dem KH für dieselbe NTM Leistung?
  - Welche Kosten entstehen dem KH daraus bzgl. der Versorgung und Diagnose?
  - Können Sie die einzelnen Posten beziffern?
- Wenn Untersuchungen nicht kostendeckend sind: Wie behilft sich die Klinik hier aktuell?
  - Z.B. durch eine Mischkalkulation?
  - Eine Abrechnung unter anderen ICD-10 Codes, z.B. für pulmonale Komorbiditäten?
    - Welche?
  - Stationäre Aufnahme von Patienten anstatt einer ambulanten Untersuchung?
    - Was wäre hierfür die Voraussetzungen bei einem NTM Patienten?
    - Patientenhistorie, Komorbiditäten? Andere?
- Deckt das vorhandene Budget die Kosten für ambulante (NTM) Patienten?
- Würde eine Budgetkürzung zu Kürzungen in der Diagnostik bzw. der Therapie führen?
- Insgesamt betrachtet, könnte eine stationäre Aufnahme der NTM Patienten die Kostensituation verbessern?
  - Auch hinsichtlich, dass NTM Patienten oft mit einer Komorbidität in das KH kommen und zusätzlich behandelt werden (COPD etc.)
  - Anregungen zu einer möglichen „integrativen Versorgungsoption“:
  - Sehen Sie eine Option darin, mit Krankenkassen oder NL Kollegen (Pulmologie) zu kollaborieren, um solch eine integrative Versorgungsoption für NTM auszuführen?
  - Wo sehen Sie dort konkret die Vorteile aber auch mögliche Risiken oder Probleme?

INTERVIEW GUIDE ZUR BEGLEITUNG DER OBSERVATIONEN VON NTM ZENTREN IN DEUTSCHLAND - FACHBEREICH: MIKROBIOLOGIE/ LABOR -

Vorstellung und Eröffnung des Interviews

- Vorstellung von ALCIMED; kurze Beschreibung des Projektes
- Können Sie bitte Ihre derzeitige Position kurz erläutern?
- Sind Sie die Hauptansprechperson in diesem Labor für die Bearbeitung von NTM Proben?
    - Wenn ja: Wie sieht dies konkret aus?
    - Wenn nicht: Wer ist dafür zuständig?

Probenmaterial und deren Analyse

- Wie viele Proben zur Untersuchung auf NTM bekommen Sie pro Woche / Monat bzw. pro Jahr in Ihr Labor geschickt?
- Was für Proben sind dies?
    - Sputum/ Tracheobronchialsekret, Abstriche, Biopsien, Blut, Liquor
    - Andere?
- Mit welcher Fragestellung kommen diese Proben zu Ihnen in das Labor?
    - Anfrage auf Testung von säurefesten Stäbchen allgemein?
    - Direkte Anfrage, spezifisch zur Testung auf Tuberkulose (Tb)? Auf NTM?
- Wie ist das Verhältnis der Proben, welche auf Tb oder auf NTM zu testen sind (Anfrage), bzw. die darauf positiv getestet werden (Ergebnis)?
    - Wie hat sich das Probenverhältnis in den letzten Jahren verhalten? (Sind NTM Befunde gestiegen, gleich geblieben oder gesunken?)
    - Haben sie in den letzten 2 Jahren eine Zunahme der Anforderungen zu NTM feststellen können?
        - Was könnte dafür ein möglicher Grund sein?
- Welche Untersuchungen der NTM Proben werden im ersten Durchlauf getestet?
    - z.B. Testung auf Subspezies
    - Sensitivitätstestung, Resistenztestung und Bestimmung der minimalen Hemmkonzentration (MHK)
    - Andere?
- Müssen die einzelnen Untersuchungen zu NTM von den pulmologischen Kollegen alle speziell angefragt werden?
    - Oder evtl. nur spezifische Untersuchungen? Welche?
- Gibt es Untersuchungen, welche Sie durchführen, nachdem Sie die Ergebnisse des ersten Durchlaufes erhalten haben?
    - Wenn ja, welche Untersuchungen sind dies?
    - Müssen diese dann erneut von den pulmologischen Kollegen angefragt werden?

- Benötigen Sie dafür neues Probenmaterial?

→ Können Sie in Ihrem Labor die benötigten Analysen zum Nachweis von NTM durchführen?

<u>Wenn **keine** NTM Analysen oder nur Teile der Analyse durchgeführt werden:</u>

- Falls Sie kooperieren: Mit welchem (externen) Labor kooperieren Sie für die Untersuchung von Proben mit dem Verdacht auf NTM?
    - z.B.: Forschungszentrum Borstel/Leibniz Lungenzentrum; Synlab MVZ Gauting; Labore der Limbach Gruppe; MVZ Dr. Eberhard & Partner Dortmund
    - Andere?
- Bei welchen Fragestellungen bzgl. NTM fragen Sie Leistungen eines externen Labors an?
    - Kooperieren Sie für alle NTM Untersuchungen mit den externen (Labor-) Kollegen, oder nur für besondere Fragestellungen (also für Teile der Analyse)?
    - Welche?
        - Bestimmung der Subspezies, Resistenztestung, Bestimmung der minimalen Hemmkonzentration (MHK) als quantitative Resistenzbestimmung etc.
- Übernimmt dieser externe Kooperationspartner auch Untersuchungen welche über den Nachweis von NTM hinausgeht?
    - Z.B. den Nachweis von Tuberkulose; oder andere allgemeine Laborleistungen?
- Wie haben Sie diesen Kooperationspartner gefunden?
    - Was waren für Sie die Kriterien zur Auswahl?
    - Hatten Sie eine Wahl, oder gibt es derzeit in Ihrem Umfeld nur eine limitierte Auswahl an geeigneten Laboren?
- Welche Zertifizierung/ Akkreditierung müssten Sie vorweisen, um NTM Proben zu untersuchen?
- Können Sie mir nennen, aus welchen Gründen Sie diese Zertifizierung nicht besitzen?
    - Mangel an Fach-Personal
    - Keine ausgewiesenen Labore (S2/S3 Labore)
    - Fehlende (administrative/finanzielle) Unterstützung für den Prozess der Akkreditierung?
- Wären Sie daran interessiert, eine Akkreditierung für die Untersuchung von NTM zu bekommen?

<u>Wenn NTM Analysen durchgeführt werden:</u>

- Seit wann ist Ihr Labor zertifiziert, NTM Proben zu analysieren?
- Bekommen Sie nur NTM Patientenmaterial von internen Kollegen, oder nehmen Sie auch externe Proben an?
    - Wie ist der Anteil des zugesandten Probenmaterials von internen/externen Kollegen?
- In welchem Zeitraum müssen die Proben bei Ihnen im Labor eingehen? (1-5 Tage?)
- Welche weiteren Qualitätsstandards haben Sie bzgl. der Einsendung von NTM Probenmaterial?
- Welche (konkreten) Analysemethoden wenden Sie in Ihrem Labor bezgl. der Untersuchung von NTM Proben an?

→ Je nach fachspezifischer Kompetenz, werden eher offene Fragen gestellt oder mit Hilfe von konkreten Stichpunkten unterstützt:

- Mikroskopie (Calcofluor, Gram), Kulturen (flüssig/ fest), Sequenzierung, PCR, Serologie
- Bestimmung der Subspezies und der minimalen Hemmkonzentration (MHK) als quantitative Resistenzbestimmung

      - Ab welcher Anzahl von NTM Proben würde sich die Etablierung einer validierten MHK-Testung wirtschaftlich darstellen?
  - Antibiotikaresistenzen (z.B. Makrolide, Aminoglykoside, Clofazimin)
  - Andere Untersuchungen? (z.B. MALDI-TOF)
  - Wie hoch sind die Anteile der verschiedenen Untersuchungen, die Sie durchführen?
- Welche Analysemethoden finden standardmäßig Anwendung?
  - Welche nur bei besonderer Fragestellung? Bei welcher Fragestellung?
- Sind die bei Ihnen standardmäßigen Analysen spezifisch für Ihr Labor, oder ist dies das gängige Vorgehen?
- Wenn Sie säurefeste Stäbchen diagnostizieren:
  - Wie viele sind davon Tb-positiv, bzw. wie ist das Verhältnis zwischen Tb- und NTM-positiven säurefesten Stäbchen?
  - Wie hoch ist der Anteil klinisch relevanter, pathogener NTM Keime?
    - Z.B. des MAC-Komplexes?
      - *M. avium-Complex* (MAC; schlecht behandelbar, langsamer Wachstum): *M.avium, M. intracellulare, M.* chimaera
    - Anderer pathogener Keime?
      - M. *abscessus/malmoense/xenopi/kansasii/chelonae*
      - *M. fortuitum/ chelonae/ smegmatis/ marinum*
    - Weitere: Kansasii-Gruppe (gut behandelbar)
      - *M. malmoense/ kansasii/ xenopi/ genavense, etc.*
- Welche Probenanzahl können Sie derzeit maximal hinsichtlich der NTM Testung pro Tag/ Woche untersuchen?
- Wären Sie in der Lage NTM Proben zu analysieren, wenn sich die Probenzahl drastisch erhöhen würde?
  - Könnte es dann evtl. zu einem Rückstau in der NTM Analyse kommen?
    - Was wäre dafür konkret der (Haupt-)Grund?
      - Z.B. Fehlendes Fachpersonal; Räumlichkeiten und/oder Gerätschaften
      - Andere Aspekte?

Kostendeckung

<u>Bei intern durchgeführten Analysen:</u>

- Wie rechnen Sie die Probenanalyse ab, bzw. wie setzt sich die Vergütung zusammen, hinsichtlich der Analyse von NTM Probenmaterial?
  - Welche Kosten entstehen dem Labor für die einzelnen Bestimmungen (Resistenztestung, Bestimmung der Subspezies etc.)?
  - Wenn bekannt: Wie hoch ist die Vergütung seitens der Versicherungen (GKV/PKV)?
- Rechnet es sich für Ihr Labor, wenn Sie alle angeforderten NTM Untersuchungen selbst durchführen?
  - Welche der Analysen sind für Ihr Labor kostendeckend, oder sogar gewinnbringend?
    - Wenn nicht kostendeckend gearbeitet werden kann: Warum werden diese Tests nicht ausgelagert, z.B. an übergeordnete, größere Labore?

- Gibt es unterschiedliche Vergütungsmodelle für die eingehenden Proben von ambulanten bzw. stationären Patienten?
- Ist die Vergütung gestaffelt nach Leistung?
    - Primärdiagnostik (anlegen von Kulturen, Resistenz-Testung etc.)
    - Sekundärdiagnostik
- Gibt es Unterschiede in der Kostendeckung bei Probenanalysen von GKV oder PKV Patienten?
    - Wie sehen diese genau aus?

<u>Bei Kooperation mit externen Laborpartnern:</u>

- Kommen Kosten auf Sie zu, wenn Sie mit externen Laboren kooperieren?
    - Warum genau?
    - Wer trägt diese? Die KK oder das Labor?
- Ist es ein großer Aufwand für Ihr Labor, wenn Sie Proben vorbereiten zur weiteren Versendung?
    - Wie genau sieht dieser Aufwand aus?

Allgemeine Organisationsstruktur

- Offene Frage: Was gilt es zu beachten, wenn Patientenmaterial mit Verdacht auf Mykobakterien zu Ihnen in das Labor geschickt wird?
- Inwieweit unterscheidet sich die Bearbeitung und Analyse von NTM und Tb Material zu anderen Proben?
    - Akkreditierung/ Zertifizierung
    - Spezifisch geschultes Fachpersonal
    - Labore mit S2/S3 Zulassung
    - Sehr kosten- und/oder zeitintensive Bearbeitung
- Gibt es bei Ihnen ein intern verwendetes Erfassungssystem (z.B. Software System) für NTM Proben, welches Ihnen hilft, Wiederholungsproben oder Verläufe zu erfassen?
    - Wie sieht dieses Erfassungssystem konkret aus?
    - Welche Informationen bekommen Sie dort zu der Patientenhistorie?
    - Sind dies rein mikrobiologische Details?
    - Werden Komorbiditäten aufgelistet bzw. klinische Verläufe?
- Zur Gewinnung von Patientenproben:
    - Haben Sie eine Anleitung für Patienten zur Gewinnung von Sputumproben?
    - Wer kümmert sich darum bzw. wer gibt diese Anweisungen an den Patienten weiter?
    - Gibt es dazu regelmäßige Schulungen des Personals (Personal/Ärzte/Schwestern)?

Kommunikation mit anderen Fachabteilungen

- Welche Kommunikation existiert zwischen den Kollegen im Labor und den internen bzw. externen Zuweisern?
    - In welcher Form passiert dies? Per Email/ Telefon?
    - Ist dies ausreichend bzw. funktioniert dies gut?
    - Welche Fragen kommen häufiger als Rückmeldung an Ihr Labor?

    - Bei unklarer Aufgabenstellung oder Rückfragen: Ist die Kommunikation eher einseitig, d.h. geht diese z.B. nur von Ihnen aus?
- Fänden Sie eine intensivere Kommunikation mit klinischen Kollegen sinnvoll?
    - Was wäre Ihr Beitrag dazu?
- Bekommen Sie klinische Daten (z.B. zur Patientenhistorie) über die Patienten, von denen Sie Proben analysieren?
    - Wenn nicht: Werden diese nachträglich angefragt?
    - Wofür benötigen Sie weitere Patientendaten? (z.B. zur Befundung?)
- Wenn zutreffend: Wie sieht die Kommunikation mit den Kollegen der externen Labore aus, welche die NTM Untersuchungen durchführen?
    - Gibt es dort einen Austausch bei bestimmten Fragestellungen?
    - Welche Fragestellungen? Wie geschieht dies? (Telefon, Email etc.)
- Wird die Kommunikation im Gesamten als ausreichend wahrgenommen?
    - Haben sie offene Bedürfnisse in dem Bezug auf die Kommunikation mit Ihren Kollegen zum Thema NTM? Wie sehen diese aus?
- Wenn Sie Ihre NTM Befunde erstellen: Welche Informationen werden dort mitgeteilt?
- Geben Sie Hinweise auf den klinischen Stellenwert oder die Infektiösität der verschiedenen Keime?
    - Z.B. zum *M. abscessus Complex* (MAC) oder *M. gordonae*? Andere?
- Geben Sie den Klinikern Hinweise auf die deutschen Empfehlungen zur Behandlung von NTM?
    - Z.B. hinweisend auf die aktuellen diagnostischen Kriterien und Behandlungsstrategien bei pulmonalen Infektionen durch NTM im „Statement der American Thoracic Society (ATS)“?
- Weisen Sie auf eine nötige Nachverfolgung und auf das Einsenden erneuter Sputumproben nach bestimmten Zeiträumen oder bei Nichtansprechen der Therapie hin?
    - Wenn nicht: Was ist dafür der Grund?
- Gibt das Labor Therapieempfehlungen an die pulmologischen Kollegen ab?
    - Wenn ja: Wie sieht eine Empfehlung aus? Ist diese unterfüttert mit Literatur?
    - Sind Ihre Empfehlungen einmalig, oder geben Sie Empfehlungen auch im Verlauf der Therapie und ggf. bei Therapieumstellung?
    - Gibt es aktive Anfragen seitens der Kliniker bzgl. der Therapieempfehlungen?

INTERVIEW GUIDE ZUR BEGLEITUNG DER OBSERVATIONEN VON NTM ZENTREN IN DEUTSCHLAND
- FACHBEREICH: PULMOLOGIE -

Vorstellung und Eröffnung des Interviews

- Vorstellung von ALCIMED; kurze Beschreibung des Projektes
- Können Sie bitte Ihre derzeitige Position kurz erläutern?
- Inwieweit sind sie in die Diagnose, Betreuung und Management von NTM Patienten involviert?
- Welche Leitlinien kennen Sie und welchen folgen Sie derzeit in Bezug auf NTM?

Patientenzahlen

- Wie viele Patienten sehen Sie täglich in Ihrer pulmologischen Fachabteilung?
    - Wie hoch ist der Anteil ambulant behandelter Patienten bzw. solcher, die stationär aufgenommen werden?
- Wie viele Patienten sehen sie pro Monat/ Jahr zur Abklärung einer Tuberkulose Infektion?
- Wie viele Patienten sehen Sie, welche mit einer spezifischen Fragestellung zur Abklärung einer NTM Infektion zu Ihnen kommen?
    - Wird diese Anfrage weiter spezifiziert? Wie sieht diese Fragestellung konkret aus?
        - Z.B. Anfrage auf Testung von säurefesten Stäbchen allgemein?
    - Bekommen Sie klinische Vorbefunde des Patienten? Welche?
- Wie häufig sehen Sie Patienten, welche mit einer Fragestellung eines unklaren Infiltrats bzw. Rundherdes zu Ihnen kommen?
    - Bei wie vielen dieser Patienten stellt sich ein positiver NTM Befund heraus?
- Hat sich der Anteil an Tb- und NTM-diagnostizierten Patienten erhöht in den letzten Jahren?
    - Sind NTM Befunde gestiegen, gleich geblieben oder gesunken?
    - Was könnte ein möglicher Grund sein?
- Was ist derzeit die maximale Patientenzahl mit NTM, welche Sie pro Woche behandeln können?
- Angenommen, die Patientenzahl mit einer (möglichen) NTM Infektion würde sich drastisch erhöhen, wäre Ihre Fachabteilung (ggf. die Ambulanz) in der Lage dies zu bearbeiten?
    - Wenn nicht, an was würde es mangeln? (Fachpersonal; Untersuchungsräume etc.)
    - Hätte eine stark erhöhte NTM Patientenzahl weitere Auswirkungen?
        - Längere Intervalle bei Untersuchungen, längere Wartezeiten etc.
- Wie ist die Verteilung der Patienten, welche zu Ihnen in die Pulmologie kommen bzgl. des Status der Diagnose (NTM Patient; Abklärung möglicher NTM Erstdiagnose; unter NTM Behandlung; zur NTM Überwachung)?
    - Patienten mit einer NTM Vordiagnose? (<u>NTM Patient</u>)
    - Patienten mit einer Überweisung zur Abklärung einer NTM Infektion (<u>Erstdiagnose)</u>
        - Z.B. mit Verdacht auf NTM oder als Zufallsbefund wegen „unklarer Diagnose“
    - Patienten <u>unter NTM Behandlung</u>

        - Wie häufig ist Ihnen der Patient schon von früheren Untersuchungen bekannt?
    - Patienten für Kontrolluntersuchungen nach erfolgreicher NTM Behandlung (NTM Überwachung)
        - In welchem Zeitabstand werden diese Patienten einbestellt?
- In welchem Stadium der Lungenkrankheit durch NTM befindet sich der Patient, wenn Sie Ihn das erste Mal sehen?
    - Z.B. leichte, mittel-schwere oder schwere Manifestation der NTM Erkrankung in der Lunge
    - Hat sich diesbezüglich ein Trend bzgl. der Patientenzahlen in den letzten Jahren abgezeichnet? Wie genau?

Überweisungsprozesse

Offene Frage: Würden Sie mir bitte darstellen, wie die Überweisung eines NTM Patienten zu Ihnen in die Fachabteilung aussieht?

Folgende Details sollten besprochen werden:

- Durch wen werden Ihnen die NTM Patienten in Ihre Fachabteilung überwiesen?
    - Kollegen aus anderen KH?
        - Wieso werden die Patienten nicht dort behandelt?
    - NL Kollegen?
        - Aus pulmologischen Praxen, vom Hausarzt etc.?
    - Wie ist der jeweilige Anteil (NL/KH)?
- Nachdem der Patient bei Ihnen untersucht wurde, überweisen Sie diesen zurück an den Zuweiser?
    - Oder bleibt dieser Patient nach positiver NTM Diagnose in Ihrer Zuständigkeit?
    - Wer bestimmt die weiteren regelmäßigen Untersuchung (NTM Überwachung) bzw. eine mögliche NTM Therapie?
        - Ihre Fachabteilung direkt oder läuft dies über den Zuweiser?

Offene Frage:
Sehen Sie derzeit Bedarf an einer Verbesserung der NTM Patientenüberweisung? Wie sähe dieser konkret aus?

Allgemeine medizinische Abläufe (spezifische Abläufe werden unten gesondert gelistet)

- Wenn ein Patient mit Verdacht auf NTM zu Ihnen in die Klinik kommt, wie sieht der medizinische Ablauf aus?
    - Mit welcher konkreten medizinischen Fragestellung kommt dieser Patient zu Ihnen?
- Gibt es bei Ihnen standardisierte Protokolle oder einen Behandlungsplan für das Management für NTM Patienten?
    - Wenn ja, können Sie diese mit uns teilen?
    - Wenn nicht vorhanden: Wäre solch ein Behandlungsplan etc. für Ihre Fachabteilung von Nutzen? Würden Sie dies begrüßen?

- Unterscheidet sich der Ablauf, wenn der Patient mit einer Fragestellung eines unklaren Infiltrats bzw. Rundherdes zu Ihnen kommt im Gegensatz zur direkten Abklärung von NTM?
  - Wenn ja: Was sind die konkreten Unterschiede?
- Inwieweit unterscheiden sich die Untersuchungsabläufe von NTM Patienten hinsichtlich des Stadiums der NTM Diagnose?
  - Bei Erstdiagnose
  - Unter NTM Behandlung
  - Bei Routine Untersuchungen bzw. NTM Überwachung und Wiedervorstellung

Untersuchungen und Therapie:

- Ab wann wird eine Therapie bei Patienten mit NTM eingeleitet?
  - Wer ist zuständig für die Therapieeinleitung? (Zuweiser oder das KH)
  - Nach welcher Leitlinie wird therapiert?
- Wie sieht der Untersuchungsablauf von NTM Patienten am Tag der Vorstellung bei Ihnen in der pulmologischen Fachabteilung aus? Welche werden eingeleitet und wohin wird überwiesen?
  - Hinsichtlich der verschiedenen medizinischen Fragestellungen bzgl.
    - Abklärung einer wahrscheinlichen NTM Infektion (Erstdiagnose)
    - Abklärung einer unspezifischen Lungeninfektion, mit Verdacht auf NTM
    - Patienten unter NTM Behandlung
    - Patienten für Routine Untersuchungen nach erfolgreicher NTM-Behandlung (NTM Überwachung)
- Welche Proben werden je Patientengruppe für weitere Untersuchungen entnommen?
- In welchen Abständen werden Untersuchungen durchgeführt, bzw. wie oft bestellen Sie den Patienten wieder ein?
  - Erfolgt dies automatisch durch das KH oder wird dies über den Zuweiser an den Patienten kommuniziert?

Organisationsstruktur

- Hat dieses KH eine pulmologische Ambulanz?
  - Ist diese Ambulanz auch für die Versorgung von NTM Patienten zuständig?
  - Gibt es eine NTM-spezifische Ambulanz? Bestünde Interesse bzw. Nachfrage?
- Nach welchem Versorgungskonzept rechnet Ihre Fachabteilung NTM-spezifische ambulante Leistungen ab?
  - ASV (Ambulante spezialfachärztliche Versorgung; in Bayern gibt es nur 1 ASV)
    - Wenn keine ASV vorhanden ist:
      - Wieso wurde bisher keine ASV im KH beantragt?
      - Gibt es Pläne, dies zukünftig zu beantragen?
    - Gab es schon Probleme bei personengebundener ASV, wenn Mitarbeiter die Abteilung verlassen haben?
    - Wie wurden bzw. würden sie in Zukunft solche Probleme behoben bzw. beheben?
  - Persönliche Ermächtigung (PE)
    - Für welche Art von Patienten ist eine PE vorhanden?

        - Ist die Anzahl von Patienten in der PE eingeschränkt?
        - Was wären die Auswirkungen, wenn sich die Patientenzahlen (z.B. für NTM) stark erhöhen würden?
    - MVZ
    - Andere ? (Hochschulambulanz; Spezialambulanz?)
- Wenn bekannt: Welche Kodierung erhalten die erbrachten NTM Leistungen?
    - Bei NTM als Erstdiagnose?
    - Bei NTM Diagnose, mit z.B. COPD als Teil der Vorgeschichte
- Wenn bekannt: Wie viel Vergütung erzielt eine "ICD-10:A31.0" Diagnose?
    - Wenn nicht zuvor beantwortet: Wie sehen die Unterschiede aus, wenn NTM als Erst- bzw. als Zweit-Diagnose gestellt wird?
- Durch wen werden die NTM-spezifischen Leistungen bei Ihnen kodiert?
    - Erfolgt dies durch Sie oder einen Kollegen (wen?)? Gab es eine DRG Schulung?
    - Oder erfolgt dies durch Kollegen vom Controlling?
- Wenn durch diese Fachabteilung kodiert wird: Nach welchen Kriterien wird kodiert?
    - Finden alle Diagnosen eine Berücksichtigung in der Kodierung?
    - Wenn ja: Nach welcher Abstufung bezgl. der Haupt- und Nebendiagnosen?
    - Wenn nein: Warum wird nicht alles berücksichtigt?
    - Gibt es Fälle, bei der die Diagnose für NTM (A31.0) nicht angegeben bzw. kodiert wurde, obwohl es hätte so sein müssen? (NTM Patient)
        - Worin liegt da der Grund?
- Wenn bekannt: Inwieweit können mit der NTM Kodierung die Kosten der NTM Leistungen gedeckt werden?
    - Wie sehen die Unterschiede in der Vergütung bei der Abrechnung ambulanter Patienten (EBM Ziffer; ASV etc.) und stationärer Patienten (DRG Satz) aus?

- Wie sehen die Unterschiede im Management von NTM Patienten aus im Vergleich zu anderen Patienten, z.B. mit Tb?
    - Woran liegt dies?
- Kam es bisher zu Engpässen bei der Terminierung bzw. Untersuchung von NTM Patienten? Was wäre hier der limitierende Faktor bei signifikant mehr Patienten pro Woche?
    - Personalmangel
    - Andere Gründe?
- Wie sähen das NTM Patientenmanagement (Wartezeit etc.) aus, wenn sich die Patientenzahl stark erhöhen würde?
    - Was wären dann die größten Probleme? (Fachpersonal, Räumlichkeiten etc.)
- Wie lange muss ein NTM Patient durchschnittlich auf einen Termin bei Ihnen warten?

- Für die Organisation von NTM Patienten: Benutzt Ihre Fachabteilung ein Erfassungssystem (Software oder ähnliches), um Patientendaten zu extrahieren, so dass diese bei z.B. Folgeuntersuchungen schnell und einfach wiedergefunden werden können? (Anzeige & Nachverfolgen der Patientenhistorie)
    - Was für ein System ist dies?

    - Wenn keine digitale Datenorganisation stattfindet: Wie wird dann die Patientenhistorie gesammelt? Auf Papier bzw. in der Patientenakte? Ist dies ausreichend?
- Organisieren Sie oder Ihre Kollegen aus der Pulmologie regelmäßige interne Treffen zum Thema NTM?
    - Wenn ja: Was wird dort besprochen?
    - Wenn nicht: Gibt es einen Bedarf an solchen Treffen?
- Hat Ihre Fachabteilung ein „Lung Board“?
    - Wäre es möglich, in diesem multidisziplinären Team diverse NTM Patientenfälle zu besprechen?
        - Hinweis: Aufgrund der niedrigen NTM Patientenzahlen kann ein reines „NTM Board“ nur schwierig als solches alleine existieren
    - Oder haben Sie NTM Patientenfälle in andere bestehende Strukturen integriert?
        - In welche? Evtl. in ILD- (interstitielle Lungenerkrankung) oder Onkologie-Boards?

Offene Diskussion:

- Gibt es zur Organisationsstruktur Aspekte, welche verbessert werden könnten? Welche wären dies und wie sähen Ihre Lösungsansätze dazu konkret aus?

Kommunikation mit anderen Fachabteilungen

Offene Frage: Mit wem tauschen Sie sich regelmäßig aus bzgl. NTM und mit welchen Kollegen oder Fachabteilungen eher selten bis gar nicht?

Zuweiser/ externe Kollegen:

- Wie sieht die Kommunikation mit den Zuweisern aus, welche Ihnen die NTM Patienten überweisen?
    - Besteht ein Austausch, bevor der Patient persönlich bei Ihnen vorbeischaut?
    - Werden Sie vorher um Rat bezgl. NTM gebeten, bevor der Patient zu Ihnen überwiesen wird?
    - Kommt es generell zu einem regelmäßigen Austausch? Mittels welcher Kanäle?
- Bekommen Sie Informationen zur Patientenhistorie von den Zuweisern?
    - Bekommen Sie in der Regel diese notwendigen Unterlagen von den Zuweisern zeitnah und vollständig?
        - Wenn nicht zeitnah vorhanden: Gibt es Unterlagen, welche besonders häufig fehlen oder nicht aktuell genug sind?
        - Wie ist der Ablauf und Zeitaufwand, die fehlenden Unterlagen zu besorgen? (Telefon/ Email Kontakt etc.)
        - Verzögert sich der Untersuchungsablauf bzw. Behandlung bei Ihnen durch fehlende Patientenunterlagen?
- Wie sieht die Kommunikation bzgl. der Weiterleitung der NTM Befunde aus?
    - Wer wird Ihrerseits alles informiert? (NL Pulmologe, Hausarzt etc.)
- Wenn Sie die NTM Befunde erstellen:
    - Welche Informationen werden dort mitgeteilt?
    - Kommt es zu Rückmeldungen bzw. –fragen? Von wem? Wie sehen diese konkret aus?

Interne Kollegen:

- Wie sieht die Kommunikation mit (internen) KH-Kollegen aus, wie z.B. der Radiologie und dem Labor bzw. dem externen Laborpartner? (allgemein und in Bezug auf NTM)
    - Wie geschieht dies? Per Email, Telefon, persönliches Vorbeischauen in den Fachabteilungen etc.
    - Ist die Kommunikation eher einseitig, d.h. geht diese z.B. nur von Ihnen aus?
- Bekommen Sie Therapieempfehlungen von den Kollegen z.B. aus der Mikrobiologie/Labor?
    - Wie sehen diese konkret aus?
- Wird die Kommunikation im Gesamten als ausreichend wahrgenommen?
    - Haben sie offene Bedürfnisse in dem Bezug auf die Kommunikation mit Ihren Kollegen zum Thema NTM? Wenn ja, wie sähen diese aus?

Vergütung von NTM-spezifischen Leistungen

- Machen sich bei Ihnen in der Fachabteilung Unterschiede in der Vergütung fest, wenn ein NTM Patient nicht ambulant sondern stationär aufgenommen wird?
    - Wie sähe der Unterschied aus, wenn man die gleiche Untersuchung ambulant bzw. stationär abrechnet?
- Gibt es Restriktionen bezgl. der Anzahl an durchgeführten Leistungen bei ambulanten Patienten?
    - Wenn ja: Inwieweit hat dies Auswirkungen auf NTM Patienten?
- Gibt es Leistungen bei Ihnen, welche besonders schlecht vergütet werden? Welche?
- Gibt es einen Unterschied bei der Vergütung von GKV und PKV Patienten? Wie sieht dieser genau aus?

Damit alle Aspekte der NTM Behandlung besprochen werden, sind unten stehend Details gelistet. Der Arzt wird aufgefordert die Behandlung zu erläutern (siehe „Allgemeine medizinische Abläufe"); wenn Behandlungsaspekte nicht beschrieben werden, wird seitens ALCIMED nachgefragt.
Bei aktuellen Problemen wird detailliert nach Gründen und konkreten Verbesserungs-vorschlägen nachgefragt.

Vorbereitung

- Was soll bei der Patientenaufnahme zur Abklärung eingeleitet werden, und wie geschieht dies?
    - Liegt eine vollständige Akte und Patientenhistorie vor?
        - Durch wen wird diese gesichtet? (Arzt, Assistent, Schwester etc.)
    - Ist immer ein Arztbrief des Zuweisers vorhanden? Wird dieser ggf. angefordert oder als Voraussetzung für die Behandlung eingefordert?
    - Wie sieht der Ablauf bei ambulanter o. stationärer Aufnahme des Patienten aus?
        - Wer ist für die Terminierung und Untersuchungsplanung zuständig?
        - Können Sie mir Laufzettel bzw. U-Pläne zeigen?
        - Werden z.B. Inhalatoren etc. gesichtet und erfasst? Wie geschieht dies?
    - Liegt dem Zentrum eine Arbeitsanweisung für Patienten mit NTM vor? Ist eine „Checkliste" für den Ablauf bei Patienten mit NTM vorhanden?
    - Gibt es hier eine Einbindung eines interdisziplinären Teams (z.B. Radiologen, Infektiologen, Atemtherapeuten, Schwestern etc.) zu Beginn oder auch im Verlauf?

Anamnese und Befunderstellung:

- Wie hoch ist der Zeitaufwand der Anamneseerhebung bzw. der Vervollständigung der Patientenakten?
    - Durch wen geschieht dies? (Arzt, Assistent etc.)
- Werden Grunderkrankungen des Patienten übernommen oder erneut überprüft? (z.B. BE, CF, Tb (Historie), Asthma, HIV, Hepatitis)
- Wie wird der Status bzw. die Erfassung folgender Daten durchgeführt:
    - Medikamente und Ko-Medikation (Immunsuppressiva, Antibiotika-Unverträglichkeiten etc.)
- Wenn nicht schon vorher abgeklärt: Was sind die notwendigen Untersuchungen bei NTM Patienten? (siehe auch Absatz „Allgemeine medizinische Abläufe")

Besprechung der Befunde

- Ab wann wird eine NTM Therapie eingeleitet? Wer entscheidet dies und wer führt diese durch?
- Welche Therapien werden eingeleitet? (Welche Leitlinien bzw. Empfehlungen?)
- Welche Informationen bzw. Befunde werden an den Zuweiser weitergeleitet? Wird auch eine Anleitung der Therapie-Überwachung weitergeleitet?
- Bekommt der Patient eine Anleitung zur häuslichen Atemtherapie oder Bronchialtoilette bzw. Sputumgewinnung?
    - Wer ist für Rückfragen der Patienten zuständig?
    - Gibt es ein Aufklärungsgespräch für den Patienten? Wer führt dieses durch?
    - Gibt es eine Notfall Telefonnummer für Patienten?

Entlassung aus der pulmologischen Fachabteilung

- Bei Erstdiagnose: Gibt es für den Patienten eine automatische Wiedervorstellung bzw. Einbestellung, wenn der Keim identifiziert wurde?
  - Nur dies nur obligat bei bestimmten Keimen? Bei welchen?
- Wie läuft die Einbestellung des Patienten ab?
  - Ist der Zuweiser für die Einbestellung zuständig?
  - Wie engmaschig, bzw. in welchen Kontrollabstände wird der Patient wieder einbestellt?
  - Gibt es ein Empfehlungsschreiben an den Zuweiser?
- Wenn der Patient sich zur Verlaufskontrolle vorstellt: Welche Informationen werden dem Zuweiser mitgeteilt?
  - Dass der Keim bekannt ist, aber keine Therapieeinleitung stattgefunden hat? Warum?
  - Dass eine Wiedervorstellung obligat ist bei klinisch relevanten Keimen. Wie sind dann die Abstände der Kontrollen?
  - Dass eine Wiedervorstellung bei klinischer Verschlechterung stattfinden sollte
- Gibt es vor Entlassung des Patienten eine Abschlussbesprechung?
  - Wie sieht diese aus? Was wird dort besprochen?
  - Wann findet diese statt? Nach Beendigung der Therapie (12-18 Monaten)?

Wiedervorstellung von NTM Patienten

- Verlaufskontrollen: In welchem Abstand erfolgt die Wiedervorstellung des Patienten?
  - Alle 3 Monate bei stabilem Verlauf?
  - Wird ein Verlaufs-CT zwischen Monat 6-9 angeordnet?
  - Oder gibt es eine engmaschigere Kontrolle, z.B. alle 6 Wochen?
- Welche Untersuchungen werden regelmäßig durchgeführt?
  - Lungenfunktionstest; Sputum; HNO; EKG, Labor, CT, Augen etc.
  - Wo werden diese Untersuchungen durchgeführt?
- Wie sieht die Kontrolle aus bzw. welche Maßnahmen werden eingeleitet, wenn eine klinische Verschlechterung (klinische Symptomatik) beim Patienten eintritt?
  - Z.B bei persistierenden Symptomen, wie Husten und Erschöpfungserscheinungen
  - Wird eine Resistenztestung durchgeführt?
- Wie sieht eine Unterstützung bei Nebenwirkungen oder Resistenzen aus?
  - Wie sähe ein Alternativplan aus?
- Gibt es einen Austausch über ambulante Maßnahmen mit den Zuweisern oder anderen Kollegen bzgl. einer „Home Therapie"?
  - Gibt es derzeit eine Home-Therapie? (neben der Insmed Home-Therapie)

Patientenkommunikation bei Nebenwirkungen oder Problemen:

- Bei klinischer Symptomatik: Wie kann sich der Patient mit Ihnen in Verbindung setzen?
  - Per Anruf? Über den Zuweiser?
- Wie sieht die Motivation der Patienten aus, regelmäßig zu Kontrolluntersuchungen zu kommen?
  - Bzgl. der Einnahme von Medikamenten?
  - Kommunikation bei Verschlechterung der Gesundheit?
- Gibt es bei Ihnen im KH einen psychosozialen Dienst für NTM Patienten?
  - Wird dieser oft non NTM Patienten genutzt?

<u>INTERVIEW GUIDE ZUR BEGLEITUNG DER OBSERVATIONEN VON NTM ZENTREN IN DEUTSCHLAND</u>

<u>- FACHBEREICH: RADIOLOGIE -</u>

Vorstellung und Eröffnung des Interviews

- Vorstellung von ALCIMED; kurze Beschreibung des Projektes
- Können Sie bitte Ihre derzeitige Position kurz erläutern?
- Ist Ihnen NTM, also die nicht-tuberkulösen Mykobakterien, ein Begriff?
- Wie tief ist Ihnen NTM bekannt in der Diagnose, Behandlung, Mortalität?
- Welche Leitlinien kennen bzw. welchen folgen Sie derzeit in Bezug auf NTM?

Patientenzahlen und Überweisungsprozesse

- Wie viele Patienten zur Untersuchung auf NTM sehen Sie pro Monat bzw. pro Jahr in Ihrer radiologischen Abteilung?

→ Wenn diese Frage nicht klar zu beantworten ist:

- Wie häufig sehen Sie Patienten, welche mit einer Fragestellung eines unklaren Infiltrats bzw. Rundherdes zu Ihnen kommen?
    - Bei wie vielen dieser Patienten stellt sich ein positiver NTM Befund heraus?
- Wie ist die Verteilung der Patienten (NTM Patient; Abklärung möglicher NTM-Erstdiagnose; unter NTM Behandlung; zur NTM Überwachung) bei Ihnen, welche eine radiologische Untersuchung benötigen?
    - Wie viele Patienten kommen zu Ihnen mit einer NTM Vordiagnose? (<u>NTM Patient</u>)
    - Patientenüberweisung bei möglicher <u>Erstdiagnose</u>
        - Wie viele Patienten werden überwiesen, die sich mit dem Verdacht auf NTM vorstellen?
        - Wie viele Patienten werden mit einem Zufallsbefund wegen „unklarer Diagnose" untersucht?
    - Patienten <u>unter NTM Behandlung</u>
        - Wie viele Patienten werden überwiesen, welche eine Verlaufs-CT Anforderung haben?
        - Ist Ihnen der Patient schon von früheren Untersuchungen bekannt, wenn ein Verlaufs-CT angefordert wird?
        - Wie häufig kommen diese Patienten zu Ihnen in die Fachabteilung?
    - Patienten für Routine Untersuchungen nach erfolgreicher NTM Behandlung (<u>NTM Überwachung</u>)
        - In welchem Zeitabstand werden routinemäßige radiologische Untersuchungen durchgeführt?
- In welchem Stadium der Lungenkrankheit durch NTM befindet sich der Patient, wenn Sie Ihn das erste Mal sehen?
    - Z.B. leichte, mittel-schwere oder schwere Manifestation der Erkrankung in der Lunge

- Wer überweist die Patienten zu Ihnen in die Fachabteilung?
  - Sind dies ausschließlich interne pulmologische Kollegen?
  - Oder kommen auch regelmäßig Überweisungen von externen Kollegen?
  - Wie ist die Verteilung der Patientenzahlen, welche von intern/extern zugewiesen werden?
- Im Hinblick auf NTM, welche Vorinformation der Anamnese (klinische Angaben) ist für eine effiziente Befundung der CT-Bilder für Sie nötig?
- Bekommen Sie in der Regel diese notwendigen Unterlagen von den Zuweisern zeitnah und vollständig?
  - Wenn nicht zeitnah vorhanden: Gibt es Unterlagen, welche besonders häufig fehlen oder nicht aktuell genug sind?
  - Wie ist der Ablauf und Zeitaufwand, die fehlenden Unterlagen zu besorgen? (Telefon, Email Kontakt etc.)
  - Verzögert sich der radiologische Untersuchungsablauf bei Ihnen durch fehlende Patientenunterlagen?

Offene Frage:

- Gibt es derzeit (weitere) Probleme im Überweisungsprozess? Wenn ja: Welche wären dies?

Allgemeine medizinische Abläufe

- In wie weit unterscheiden sich die Untersuchungsabläufe von NTM Patienten hinsichtlich des Stadiums der NTM Diagnose?
  - Bei Erstdiagnose
  - Unter NTM Behandlung
  - Bei Routine Untersuchungen bzw. NTM Überwachung und Wiedervorstellung
- Welche Untersuchungen werden bei diesen NTM Patienten durchgeführt?
  - CT-, native Röntgen-Untersuchungen etc.
- In welchen Abständen werden diese Untersuchungen durchgeführt?
- Gibt es Fälle von NTM Patienten, die zu spät erkannt wurden und bisher unter anderen Diagnosen gelaufen sind?
  - Wenn ja, unter welchen Diagnosen?
  - Können Sie sich an solche Fälle erinnern, die progressiv verlaufen sind?
    - Sind diese zu Ihrer Zufriedenheit abgelaufen?
- Welche Kriterien gibt es Ihrer Meinung nach, um NTM von anderen Krankheiten, wie z.B. TB, zu unterscheiden?

Offene Diskussion:

- In wie weit unterscheidet sich der Ablaufplan der radiologischen Untersuchungen bei NTM Patienten und nicht-NTM Patienten?
- Wo sehen Sie derzeit Probleme hinsichtlich des Patientenmanagements? Wie sähen konkrete Ideen für eine Verbesserung aus?

- Wie sehen die Unterschiede in der Organisation der Untersuchungen von NTM Patienten im Vergleich zu anderen Patienten aus? (diese Frage ähnelt sehr der vorhergehenden Frage; hier wird es als Überleitung zu diesem Fragenblock genutzt)
  - Woran liegt dies?
- Was ist derzeit die maximale Patientenanzahl (pro Woche), die Sie in Bezug auf NTM untersuchen können?
- Kam es bisher zu Engpässen bei der Terminierung bzw. Untersuchung von NTM Patienten? Was wäre hier der limitierende Faktor?
  - Personalmangel
  - Mangel an Untersuchungsgeräten
  - Andere Gründe?
- Wie sähen die Wartezeit sowie die Organisation von NTM Patienten bei Ihnen aus, wenn sich die Patientenzahl stark erhöhen würde?
  - Was wären dann die größten Probleme? (Personal, Geräte, andere?)
- Wie lange muss ein NTM Patient durchschnittlich auf einen Termin für eine Untersuchung (welche?) bei Ihnen warten?
- Für die Organisation von NTM Patienten: Benutzt Ihre Fachabteilung ein Erfassungssystem (Software oder ähnliches), um Patientendaten zu extrahieren, so dass diese bei z.B. Folgeuntersuchungen schnell und einfach wiedergefunden werden können? (Anzeige & Nachverfolgen der Patientenhistorie)
  - Was für ein System ist dies?
  - Wenn keine digitale Datenorganisation stattfindet: Wie wird dann die Patientenhistorie gesammelt? Auf Papier bzw. in der Patientenakte? Ist dies ausreichend?
- Nehmen Sie (oder andere Kollegen der Radiologie) an regelmäßigen internen Treffen der Pulmologie zum Thema von NTM (oder zu anderen pulmonalen Themen?) teil?
  - Wenn ja: Was wird dort besprochen?
  - Wenn nicht: Würden Sie gerne an solchen interdisziplinären Treffen teilnehmen?
  - Ist die Teilnahme für Sie speziell in der Behandlung von NTM Patienten wichtig?
- Hat Ihre Fachabteilung ein „Lung Board“?
  - Wäre es möglich in diesem multidisziplinären Team diverse NTM Patientenfälle zu besprechen?
    - Hinweis: Aufgrund der niedrigen NTM Patientenzahlen kann ein reines „NTM Board“ nur schwierig als solches alleine existieren
  - Oder können NTM Patientenfälle in andere bestehende Strukturen integriert werden?
    - Evtl. in ILD- (interstitielle Lungenerkrankung) oder Onkologie-Boards?

Offene Diskussion:

- Gibt es zur Organisationsstruktur Aspekte, welche verbessert werden könnten? Welche wären dies und wie sähen Ihre Lösungsansätze dazu konkret aus?

Kommunikation mit anderen Fachabteilungen

- Wie sieht die Kommunikation mit internen und externen Kollegen aus (allgemein und in Bezug auf NTM)?
    - Wie geschieht dies? Per Email, Telefon, persönliches Vorbeischauen in den Fachabteilungen etc.
    - Ist die Kommunikation eher einseitig, d.h. geht diese z.B. nur von Ihnen aus?
- Wird die Kommunikation im Gesamten als ausreichend wahrgenommen?
    - Haben sie offene Bedürfnisse in dem Bezug auf die Kommunikation mit Ihren Kollegen zum Thema NTM? Wenn ja, wie sehen diese aus?
- Wenn Sie Ihre NTM Befunde erstellen: Welche Informationen werden dort mitgeteilt?
    - Wird der Befund deskriptiv oder auch mit Hinweis auf NTM verfasst und möglicherweise sogar mit der Notwendigkeit aktiv zu werden?
    - Wenn Sie ggf. einen Verdacht auf eine mögliche NTM Aktivität beschreiben: Mit welcher Konsequenz geschieht dies?
    - Empfehlen Sie eine Nachverfolgung? Wenn ja: In welchen Abständen?
- Gibt es Rückmeldung seitens der pulmologischen Kollegen, ob ein Patient tatsächlich positiv auf NTM diagnostiziert wurde?
    - Gibt es bzgl. NTM generell eine Rückkopplung mit den klinischen Kollegen?
    - Würden Sie dies begrüßen?

Vergütung von NTM-spezifischen Untersuchungen

- Gibt es einen Unterschied in der Vergütung der radiologischen Leistungen, wenn ein NTM Patient nicht <u>ambulant sondern stationär</u> aufgenommen werden würde?
    - Wie sähe der Unterschied aus, wenn man die gleiche Untersuchung ambulant bzw. stationär abrechnet?
        - Anm.: Radiologische Untersuchungen werden bei stationär aufgenommenen Patienten über den DRG Satz abgerechnet
        - Ambulante Abrechnungen über EBM Ziffer, bzw. andere Rechtsformen wie PE, ASV, MVZ etc.
- Für <u>ambulante Patienten</u>, werden NTM-spezifische Leistungen aus einem gemeinsamen „Topf“ für Leistungen von Patienten aus der Pulmologie abgerechnet?
- Gibt es Restriktionen bezgl. der Anzahl an durchgeführten Leistungen bei <u>ambulanten Patienten</u>?
    - Wenn ja: Inwieweit hat dies Auswirkungen auf NTM Patienten?
- Gibt es radiologische Untersuchungen (auch außerhalb NTM-relevanter Leistungen), welche besonders schlecht vergütet werden? Welche?
    - Haben Sie von der KH-Administration Rückmeldung bekommen (evtl. auch „Druck“ bekommen), weniger solcher Untersuchungen durchzuführen?
    - Hat diese Restriktion auch einen Einfluss auf Leistungen für NTM Patienten?
- Gibt es einen Unterschied bei der Vergütung von GKV und PKV Patienten? Wie sieht dieser genau aus?
